# Allicin Alleviates Reticuloendotheliosis Virus-Induced Immunosuppression *via* ERK/Mitogen-Activated Protein Kinase Pathway in Specific Pathogen-Free Chickens

**DOI:** 10.3389/fimmu.2017.01856

**Published:** 2017-12-22

**Authors:** Liyuan Wang, Hongchao Jiao, Jingpeng Zhao, Xiaojuan Wang, Shuhong Sun, Hai Lin

**Affiliations:** ^1^Poultry Oncogenic Virus Research Laboratory, College of Animal Science and Veterinary Medicine, Shandong Agricultural University, Shandong Key Lab for Animal Biotechnology and Disease Control, Tai’an, China; ^2^Department of Animal Science, College of Animal Science and Veterinary Medicine, Shandong Agricultural University, Shandong Key Lab for Animal Biotechnology and Disease Control, Tai’an, China

**Keywords:** reticuloendotheliosis virus, allicin, mitogen-activated protein kinase, nuclear factor kappa B p65, oxidative stress

## Abstract

Reticuloendotheliosis virus (REV), a gammaretrovirus in the *Retroviridae* family, causes an immunosuppressive, oncogenic, and runting–stunting syndrome in multiple avian hosts. Allicin, the main effective component of garlic, has a broad spectrum of pharmacological properties. The hypothesis that allicin could relieve REV-induced immune dysfunction was investigated *in vivo* and *in vitro* in the present study. The results showed that dietary allicin supplementation ameliorated REV-induced dysplasia and immune dysfunction in REV-infected chickens. Compared with the control groups, REV infection promoted the expression of inflammatory cytokines including *interleukin (IL)-1β, IL-6, IL-10, interferon (IFN)-*γ, and *tumor necrosis factor-α (TNF-*α*)*, whereas, allicin reversed these changes induced by REV infection. The decreased levels of *IFN-*α, *IFN-*β, and *IL-2* were observed in REV-infected chickens, which were significantly improved by allicin. Allicin suppressed the REV-induced high expression of toll-like receptors (TLRs) as well as *melanoma differentiation-associated gene 5 (MDA5)* and the activation of mitogen-activated protein kinase (MAPK) and the nuclear factor kappa B p65. REV stimulated the phosphorylation of JNK, ERK, and p38, the downstream key signaling molecules of MAPK pathway, while allicin retarded the augmented phosphorylation level induced by REV infection. The decreased phosphorylation level of ERK was associated with REV replication, suggesting that ERK signaling is involved in REV replication, and allicin can alleviate the REV-induced immune dysfunction by inhibiting the activation of ERK. In addition, REV infection induced oxidative damage in thymus and spleen, whereas allicin treatment significantly decreased the oxidative stress induced by REV infection, suggesting that the antioxidant effect of allicin should be at least partially responsible for the harmful effect of REV infection. In conclusion, the findings suggest that allicin alleviates the inflammation and oxidative damage caused by REV infection and exerts the potential anti-REV effect by blocking the ERK/MAPK pathway.

## Introduction

Reticuloendotheliosis virus (REV) is an oncogenic and immunosuppressive retrovirus, belonging to the family *Retroviridae*, specifically gammaretroviruses in the same genus as mammalian type C retroviruses (1). REV infection attacks the lymphocytes and endotheliocyte to induce immunosuppression and increases the possibility of lymphoma, reticuloendothelial sarcomas, and various non-neoplastic syndromes such as runting and anemia in multiple avian hosts (2, 3). Additionally, it is reported that REV can be combined into a number of genomes of attenuated vaccines such as Marek’s disease virus (MDV)-attenuated virus vaccine (4, 5). Recently, REV whole genome was amplified from a fowl pox virus (FPV)-attenuated virus (6), representing potential dangers to the poultry industry. However, the potential mechanisms of REV-induced immune dysfunction were still unclear, and there is no effective practical measure in the control of REV infection in chickens. It is known that MDV, another oncogenic virus in chicken, induces the overexpression of cytokines, interleukin (IL)-1*β* and IL-6, which are associated with the activation of inflammation (7). The involvement of cytokines in the pathogenicity of REV infection remains to be elucidated yet.

Allicin, the main effective component of garlic, can enter cells through the phospholipid membrane, exerting a broad spectrum of pharmacological properties. A wide range of microorganisms including bacteria, fungi, protozoa, and viruses have been shown to be sensitive to allicin (8). Moreover, allicin has an immunomodulatory function in suppressing the release of pro-inflammatory cytokines such as IL-6 and tumor necrosis factor-α (TNF-α) (9, 10). In addition, allicin can modulate the nuclear factor kappa B (NF-κB) transcription and DNA binding activity and suppress the expression of NF-κB-mediated inflammatory target genes (11). Moreover, allicin is rich in selenium and sulfur, which can interact with intracellular thiol compounds to play an antioxidant effect (12). Our previous study has demonstrated that allicin (300 mg/kg) could improve the immune function of chickens (13). Hence, we hypothesized that allicin may reverse REV-induced immune dysfunction in chickens.

In the present study, we aimed to determine the effect of allicin on immunosuppression induced by REV infection in chickens. The experimental chickens were fed a diet supplemented with allicin and were subjected to REV inoculation. Body weight was measured to evaluate the effect of REV infection on the growth of chickens. The index of immune organs and transcriptional levels of inflammatory cytokines were determined to estimate the immune status post REV infection. The involvement of signaling pathways such as MAPK in REV infection was investigated in REV-infected chickens and in lymphocyte separated from spleen. The oxidative damage of immune organs was measured as well in REV-challenged chickens.

## Materials and Methods

### Virus

The REV SNV-C5 strain was isolated from a flock of commercial layer chickens (Jiangsu, China) and was stored at our laboratory. The virus was propagated on a monolayer of primary chicken fibroblast cells (CEFs) prepared from 10-day-old special pathogen-free (SPF) chicken embryos (Sais, China).

### Animals and Experimental Design

A total of 240 1-day-old SPF White Leghorn chickens (Sais) were divided into 6 treatment groups of 40 birds per treatment: (i) control group; (ii) REV group (basal diets); (iii) 150 mg/kg allicin (Jiayijia Ltd., Weifang, China) group; (iv) 300 mg/kg allicin group; (v) REV + 300 mg/kg allicin group; and (vi) REV + 600 mg/kg allicin group. The REV-infected groups [(ii), (v), and (vi)] were subjected to REV (SNV-C5 strain) intraperitoneal inoculation (100 TCID_50_/0.2 ml) at 7 days of age, and the other three groups [(i), (iii), and (iv)] were treated with saline. The chickens in REV-infected groups were housed separately from the mock ones with the same rearing facility and similar environment.

Eight chickens were randomly selected from each group at 2, 3, 4, and 5 weeks post REV infection. The immune organs including thymuses, spleens, and bursas were collected and weighed. After measurement, the tissue samples from the four following groups were used for further analysis: control, REV group, 300 mg/kg allicin group, and REV + 300 mg/kg allicin group. The samples of immune organs were stored at −80°C for mRNA and Western blot analysis. Serum samples were collected and centrifuged at 3,000 × *g* for 10 min and were stored at −80°C for further analysis.

### Cell Culture, Virus Infection, Activator, and Inhibitor of MAPK Treatments

The lymphocytes were separated from clinically healthy SPF chickens. The spleens were removed and collected in a sterile wire sieve over a Petri dish half filled with media. The spleens were gently pressed through the 400 mesh wire screen using a plunger of the syringe to remove connective tissues and the screen was further rinsed with fresh media. The cell suspension was transferred into a 15 mL tube containing 3 mL chicken lymphocyte separation medium (TBD, China) and centrifuged at 3,000 rpm for 15 min to remove the erythrocytes and splenocyte (we could not find an effective reagent to remove erythrocytes). The separated cells were seeded at a density of 1 × 10^7^ cells/mL. The cells were preinfected with 20 TCID_50_ REV for 1 h at 4°C, and then the cells were washed three times and cultured in RPMI1640 (Gibco, USA) supplemented with 10% fetal bovine serum (FBS; BI, Israel) at 37°C in a 5% CO_2_ atmosphere. The cell viability was determined by CCK-8 kit (Trans, China) at 6 h post 20 TCID_50_ REV infection or allicin treatment and determined at the wavelength of 450 nm.

Cells were pretreated with the ERK-specific inhibitor (PD98059, 10, 50, and 100 μM), JNK inhibitor (SP600125, 10, 50, and 100 μM), p38 inhibitor (SB203580, 10, 30, and 50 μM), and ERK activator Ceramide C6 (Santa Cruz, 10 µM) for 1 h and then were mock infected or infected by incubation with 20 TCID_50_ diluted virus stocks at 4°C for 1 h, followed by incubation at 37°C and 5% CO_2_ for different time. The inhibitors were obtained from Beyotime (China).

### Real-time Quantitative PCR Analysis

Total RNA from immune organs, including thymuses, spleens, and bursas, was prepared by the acid phenol method using Trizol reagent (Invitrogen, USA) according to the manufacturer’s instructions, followed by cDNA synthesis of mRNA using the transcriptor first-strand cDNA synthesis kit (Roche, China) and amplification by qPCR with FastStart Universal SYBR Green Master (Rox) (Roche). Primers used for qRT-PCR were designed by the NCBI Primer BLAST program and DNAMAN software and were based on published target sequences (Table 1). The mRNA level of β-actin was measured as an internal control. Thermal cycling was initiated with an activation step of 30 s at 95°C, and this step was followed by 40 cycles of 95°C for 5 s and 60°C for 30 s. Immediately after amplification, melt curve protocols were performed to ensure that primer dimers and other nonspecific products were minimized. The relative expression of the target genes was analyzed by the 2^−ΔΔCT^ method.

**Table 1:** qRT-PCR primers.

Gene	Sequence (5′–3′)
Interleukin (IL)-1β	Forward TCCTCCAGCCAGAAAGTGA
	Reverse GGTAGAAGATGAAGCGGGTC
IL-2	Forward TCTTTGGCTGTATTTCGG
	Reverse CTGGGTCTCAGTTGGTGT
IL-6	Forward CTCCTCGCCAATCTGAAGTC
	Reverse AGGCACTGAAACTCCTGGTC
IL-10	Forward CGCTGTCACCGCTTCTTCA
	Reverse TCCCGTTCTCATCCATCTTCTC
Tumor necrosis factor-α (TNF-α)	Forward CATTTGGAAGCAGCGTTTGG
	Reverse GGTTGTGGGACAGGGTAGGG
Interferon (IFN)-α	Forward GACAGCCAACGCCAAAGC
	Reverse GTCGCTGCTGTCCAAGCATT
IFN-β	Forward GCCCACACACTCCAAAACACTG
	Reverse TTGATGCTGAGGTGAGCGTTG
IFN-γ	Forward CTGACGGTGGACCTATTATTGTAG
	Reverse GTTTGATGTGCGGCTTTGA
IRF7	Forward TATCTTCCGCATCCCTTG
	Reverse GTTGGTCTTCCATTTGGC
ISG12-1	Forward TAAGGGATGGATGGCGAAG
	Reverse GCAGTATCTTTATTGTTCTCAC
TLR3	Forward GACAAACTTCACCTCTCTGGA
	Reverse CTTCCTGCTCCTTCTTATGC
TLR4	Forward GAAGGGAAGGCTGGAATAA
	Reverse GTGGGAGACAGGACAGAAGT
TLR7	Forward TCTGGACTTCTCTAACAACA
	Reverse AATCTCATTCTCATTCATCATCA
MDA5	Forward ATTCCACAGCCGCAGATTC
	Reverse CAAGATTGGCACAGATTTTCAGA
MyD88	Forward AGAGTTGGAGCAAACGGA
	Reverse TGAAATGACGACCACCATC
pol	Forward CCCCATTCATGTCCAGCTAT
	Reverse AGGGAGGAGAGGAGTGTTCC
LTR	Forward TTGHTTGAAGGCAAGCATCAG
	Reverse GAGGATAGCATCTGCCCTTT
β-actin	Forward CTGGCACCTAGCACAATGAA
	Reverse CTGCTTGCTGATCCACATCT

*The TNF-α referred to in this article is lipopolysaccharide-induced TNF-α factor*.

### Western Blot Analysis

Tissue homogenates from the thymuses and spleens were centrifuged at 12,000 × *g* and 4°C for 10 min. The protein content of the supernatants was determined using the BCA protein assay kit (Beyotime). Total protein (50 μg) was separated by SDS-PAGE and was transferred to PVDF membranes (Millipore, Merck, Germany) using a transfer apparatus (BioRad, USA). The membranes were blocked with blocking buffer (Beyotime) at room temperature for 2 h and then were incubated with anti-phospho-P38 [#4511T, anti-rabbit, Cell Signaling Technology (CST), USA], anti-P38 (#9212S, anti-rabbit, CST), anti-phospho-JNK (#4668S, anti-rabbit, CST), anti-JNK (#928, anti-rabbit, CST), anti-phospho-ERK (#9101S, anti-rabbit, CST), anti-ERK (#9102S, anti-rabbit, CST), anti-TLR-3 (NBP2-24565, anti-rabbit, NBP2-24565Novus Biologicals, USA), and anti-TLR-4 (BA1717, anti-rabbit, Boster, China) primary antibodies overnight at 4°C, followed by incubation with the corresponding horseradish peroxidase-conjugated secondary antibody (Beyotime) at 4°C for 4 h. The protein–antibody complexes were detected with the ECL Plus A and B (Beyotime), and the results were quantified using the Fusion FX software (Vilber, France).

Nuclear and cytoplasmic proteins were prepared using the nuclear and cytoplasmic protein extraction kit (Beyotime), and then the levels of NF-κB p65 (D14E12) subunit (#3033P, anti-rabbit, CST), nuclear factor erythroid 2 p45-related factor 2 (Nrf2) (ab62352, anti-rabbit; Abcam, Britain), and inhibitory protein-κBα (IκBα) (44D4) (#4812S, anti-rabbit, CST) were detected. The protein levels of tubulin (AT819, anti-rat; Beyotime) and lamin B1 (ab20396, anti-rat, Abcam) were detected as internal standards of cytoplasmic and nuclear protein, respectively.

### Determination of the Oxidative Damage Parameters

The tissues were homogenized in 0.9% NaCl solution and centrifuged at 3,000 × *g* for 5 min. The supernatants were collected to determine the antioxidant function. The method used to detect the malondialdehyde (MDA) level in this study was performed as previously described (14). The activities of antioxidizes including superoxide dismutase (SOD), catalase (CAT), glutathione peroxidase (GSH-PX), and the levels of GSH and protein carbonyls in the thymuses, spleens homogenates, and serum were determined with commercial kits (Jiancheng, China).

### Statistical Analysis

The data were expressed as mean ± SE and analyzed by one-way ANOVA with SAS software. Multiple comparisons between the groups were performed by the Tukey method. *P* < 0.05 was considered as statistically significant.

## Results

### Allicin Promotes REV-Induced Immunosuppression and Growth Inhibition

**Figure 1:**
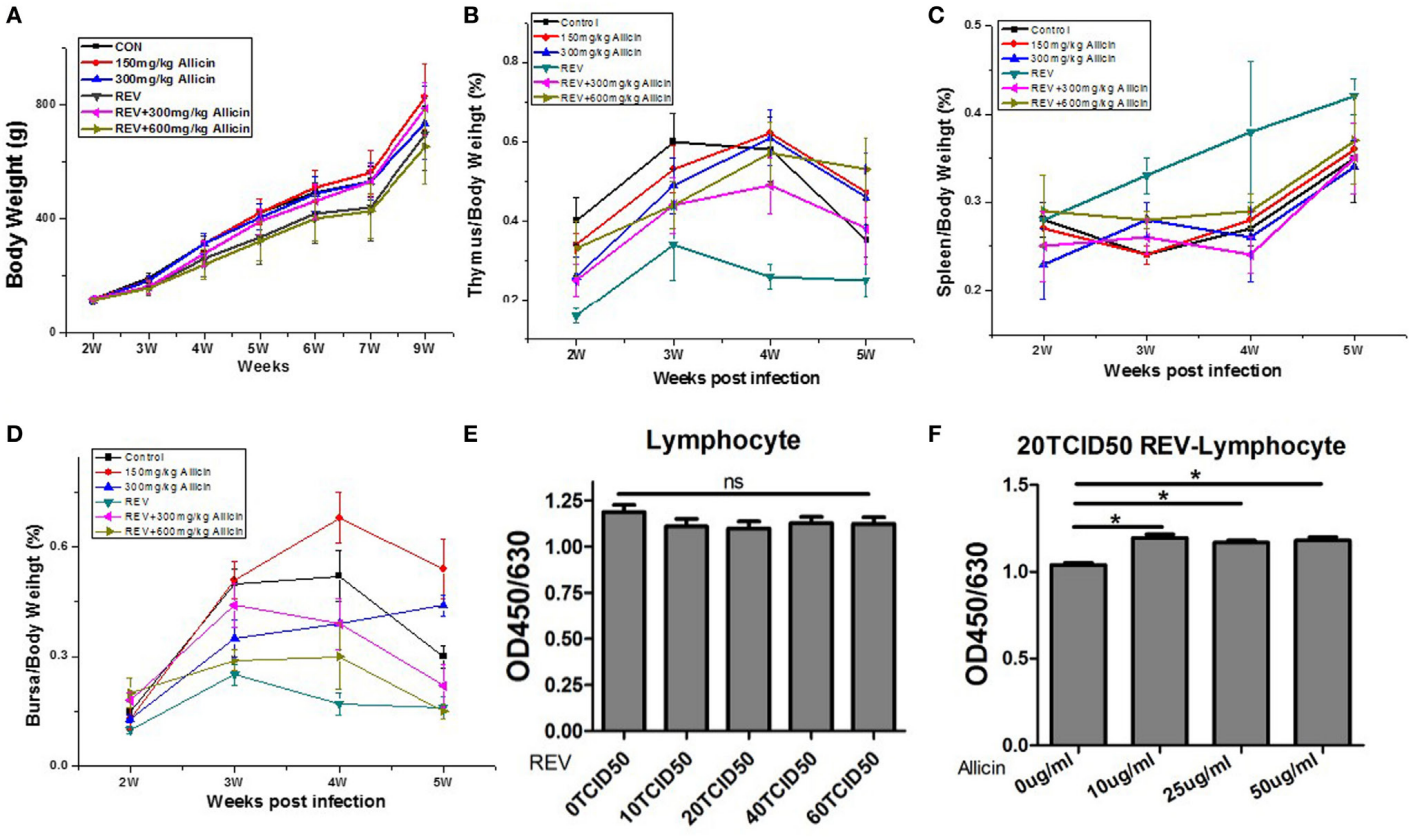
Allicin treatment improves reticuloendotheliosis virus (REV)-induced inhibition of growth and immune function. **(A)** The body weight loss observed in the REV group was improved by 300 mg/kg allicin treatment (Week 2: *n* = 40/group; Week 3: *n* = 32/group; Week 4: *n* = 24/group; Week 5: *n* = 16/group; and Weeks 6, 7, and 9: *n* = 8). **(B)** The index of thymuses (*n* = 8). **(C)** The index of spleens (*n* = 8). **(D)** The index of the bursa of Fabricius (*n* = 8). **(E)** The viability of REV-infected cells (*n* = 6). **(F)** The effect of allicin on the viability of 20 TCID_50_ REV-infected cells (*n* = 6). The values are expressed as mean ± SE.

To evaluate the effect of allicin on REV infection, the growth of body weight and immune organs were determined. REV administration induced body weight loss from the second week after infection (*P* < 0.0001, Figure 1A), compared with the control chickens. However, allicin supplement at 300 mg/kg (*P* < 0.05) rather than 600 mg/kg (*P* > 0.05) significantly ameliorated the retarded body weight in REV-infected chickens. In contrast, allicin supplementation had no detectable effect on normal chickens (*P* > 0.05). REV challenge resulted in atrophy of thymuses (*P* < 0.05) from the second week post REV infection (Figure 1B). Allicin supplementation at both 300 and 600 mg/kg levels alleviated the thymus atrophy induced by REV (*P* < 0.05). The weight of spleens in REV-infected chickens was higher than that of control and allicin group, but there was no statistically significant difference (*P* > 0.05, Figure 1C). REV decreased the weight of bursas, while allicin supplementation at 300 mg/kg level relieved the REV-induced atrophy (Figure 1D). In addition, though REV decreased the viability of lymphocyte separated from spleens in a certain extent, there was no statistically significant difference (*P* > 0.05, Figure 1E). Allicin treatment improved the proliferation of REV-infected lymphocyte (*P* < 0.05, Figure 1F). These findings indicated that allicin partially alleviated the immune dysfunction and growth inhibition induced by REV infection, and REV infection model was successfully established.

### Allicin Decreases the Expression of Inflammatory Cytokines in REV-Infected Chickens

**Figure 2:**
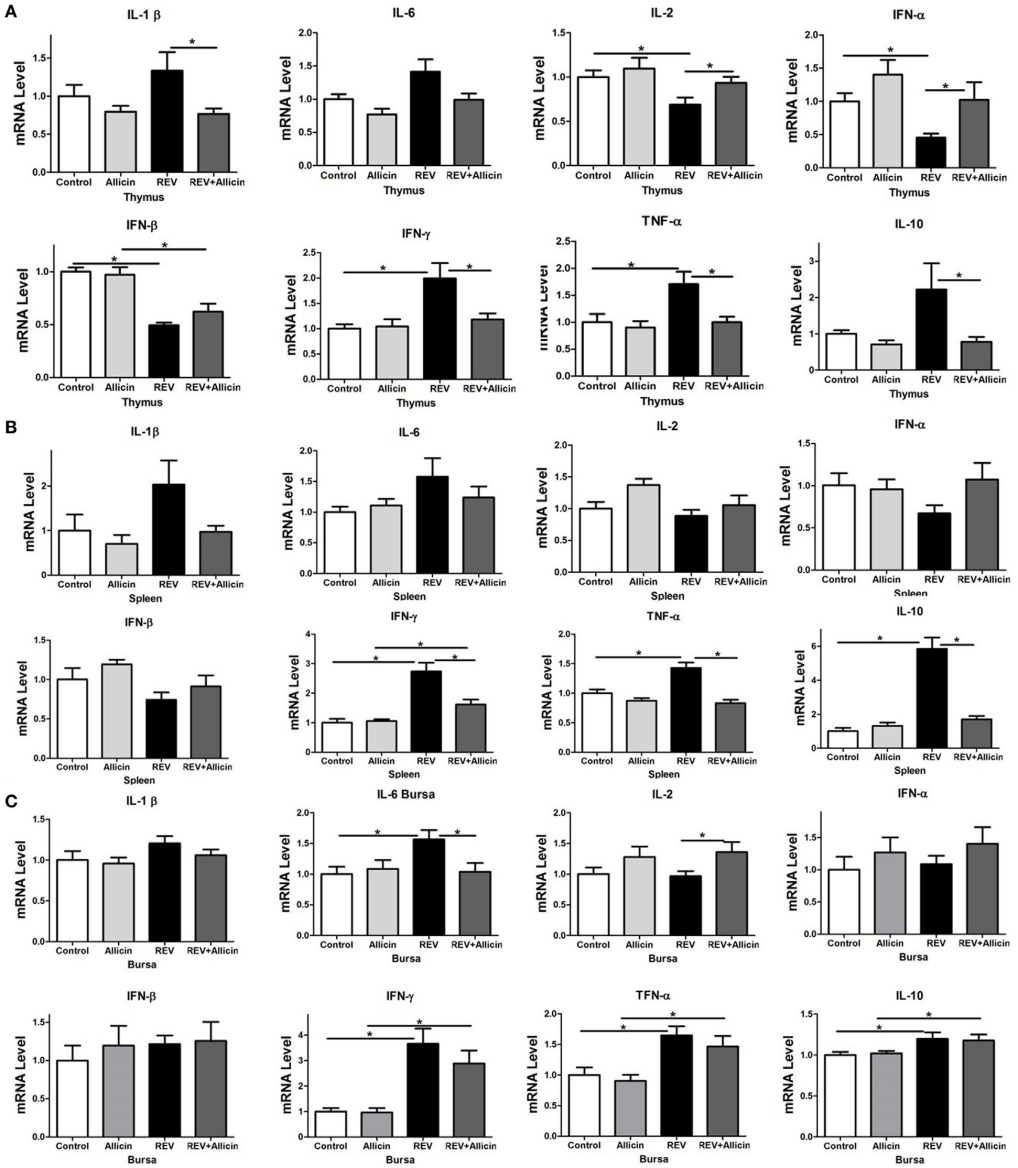
Effect of 300 mg/kg allicin on the release of inflammation-related cytokines in reticuloendotheliosis virus-infected chickens. **(A)** Transcriptional levels of cytokines in thymuses. **(B)** Transcriptional levels of cytokines in spleens. **(C)** Transcriptional levels of cytokines in bursas. All data are presented as mean ± SE (*n* = 8) (folds of the control group). “*” denotes a statistically significant difference at *P* < 0.05 compared with the other group.

To further explore the changes in the immune responses during REV infection, we measured the expression levels of cytokines in immune organs. According to the above results, we mainly detected the expression levels of cytokines in four groups of chickens including control group, 300 mg/kg allicin group, REV group, and REV + 300 mg/kg allicin group. As shown in Figure 2, we detected the upregulated levels of *IL-1*β, *INF-*γ, *IL-10*, and *TNF-*α in REV-infected thymuses (*P* < 0.05, Figure 2A) and spleens (*P* < 0.05, Figure 2B); and the expressions of *IL-6, IL-10, INF-*γ, and *TNF-*α in bursas (*P* < 0.05, Figure 2C) of REV-infected chickens were significantly increased compared to the control birds. However, allicin treatment significantly downregulated the decreased expression of the cytokines. In addition, the expression of *interferon (IFN)-*α (*P* < 0.05), *IFN-*β (*P* < 0.05) in thymus, and *IL-2* in thymuses and bursas (*P* < 0.05) (Figure 2C) were significantly decreased in REV-challenged chickens compared with the allicin treated group, and treatment with allicin increased the levels upon REV infection (*P* > 0.05). Feeding with allicin without REV infection had no significant influence on chickens compared with the control group (*P* > 0.05).

### Allicin Downregulates the Differential Expression of the Pattern Recognition Receptors (PRRs) Induced by REV Infection

**Figure 3:**
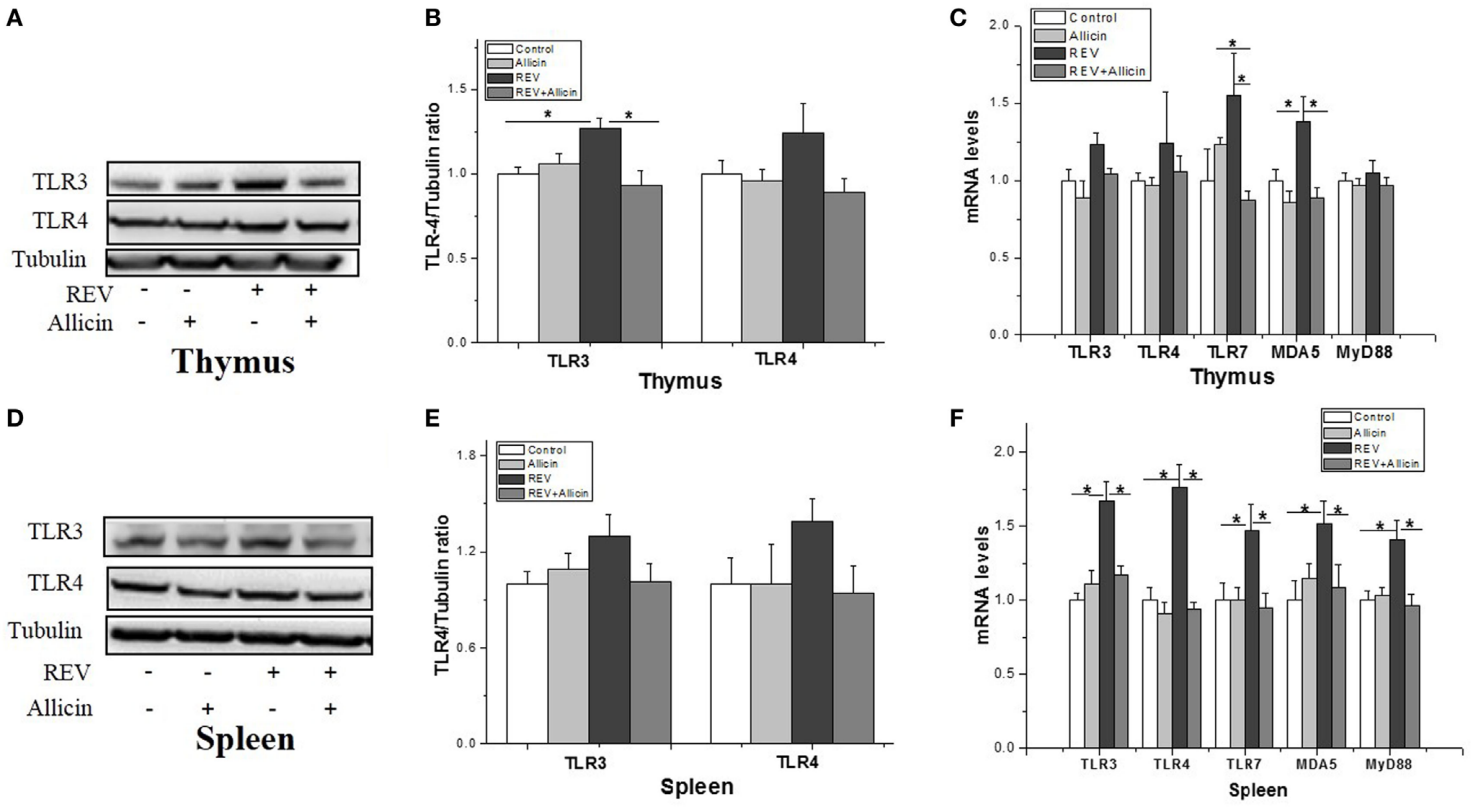
Transcriptional analysis of pattern recognition receptors (PRRs) in experimental chicks. **(A,D)** Protein levels of TLR-3 and TLR-4 in thymuses and spleens. **(B,E)** Relative level of TLR-3 and TLR-4 to tubulin. **(C,F)** Transcriptional level of *PRRs* and *myeloid differentiation factor 88 (MyD88)*. All data are presented as mean ± SE (*n* = 8) (folds of the control group). “*” denotes a statistically significant difference at *P* < 0.05 compared with the other groups.

The induction of the antiviral innate immune response depends on the recognition of pathogen-associated molecular patterns (PAMPs) of viral components by PRRs. The TLRs family emerged as key sensors that recognize viral components to regulate both innate and adaptive immunity. As observed in Figure 3, REV infection promoted the protein levels of TLR-3 rather than TLR-4 in thymuses (Figures 3A,B), whereas had no significant effect on TLR-3 and TLR-4 at the protein level in spleens (*P* > 0.05) (Figures 3D,E). The mRNA levels of TLR-3 (*P* < 0.05) and TLR-7 (*P* < 0.05) (Figure 3C) in thymuses of REV-infected chickens also were increased by REV infection. In REV-infected chickens, allicin treatment significantly reduced the protein level of TLR-3 (*P* < 0.05) in thymuses. REV infection upregulated the expression of *TLR-3* (*P* < 0.05), *TLR-4* (*P* < 0.05), and *TLR-7* (*P* < 0.05) in spleens, whereas allicin treatment significantly arrested the stimulating effect of REV on the expression of *TLR-3* (*P* < 0.05), *TLR-4* (*P* < 0.05), and *TLR-7* (*P* < 0.05) (Figure 3F). In addition, the expression of *myeloid differentiation factor 88 (MyD88)*, a key adapter molecule of the TLRs signaling pathway, and *melanoma differentiation-associated gene 5 (MDA5)* was measured. These results showed that REV infection significantly upregulated the mRNA levels of *MyD88* (*P* < 0.05) and *MDA5* in spleens (*P* < 0.05), whereas allicin treatment downregulated their expression levels in REV-infected chickens (*P* < 0.05). In thymus, the mRNA level of *MDA5* was upregulated by REV and downregulated by allicin in REV-infected chickens (*P* < 0.05, Figure 3C).

### Allicin Inhibits the REV-Activated MAPK and NF-κB Pathways

**Figure 4:**
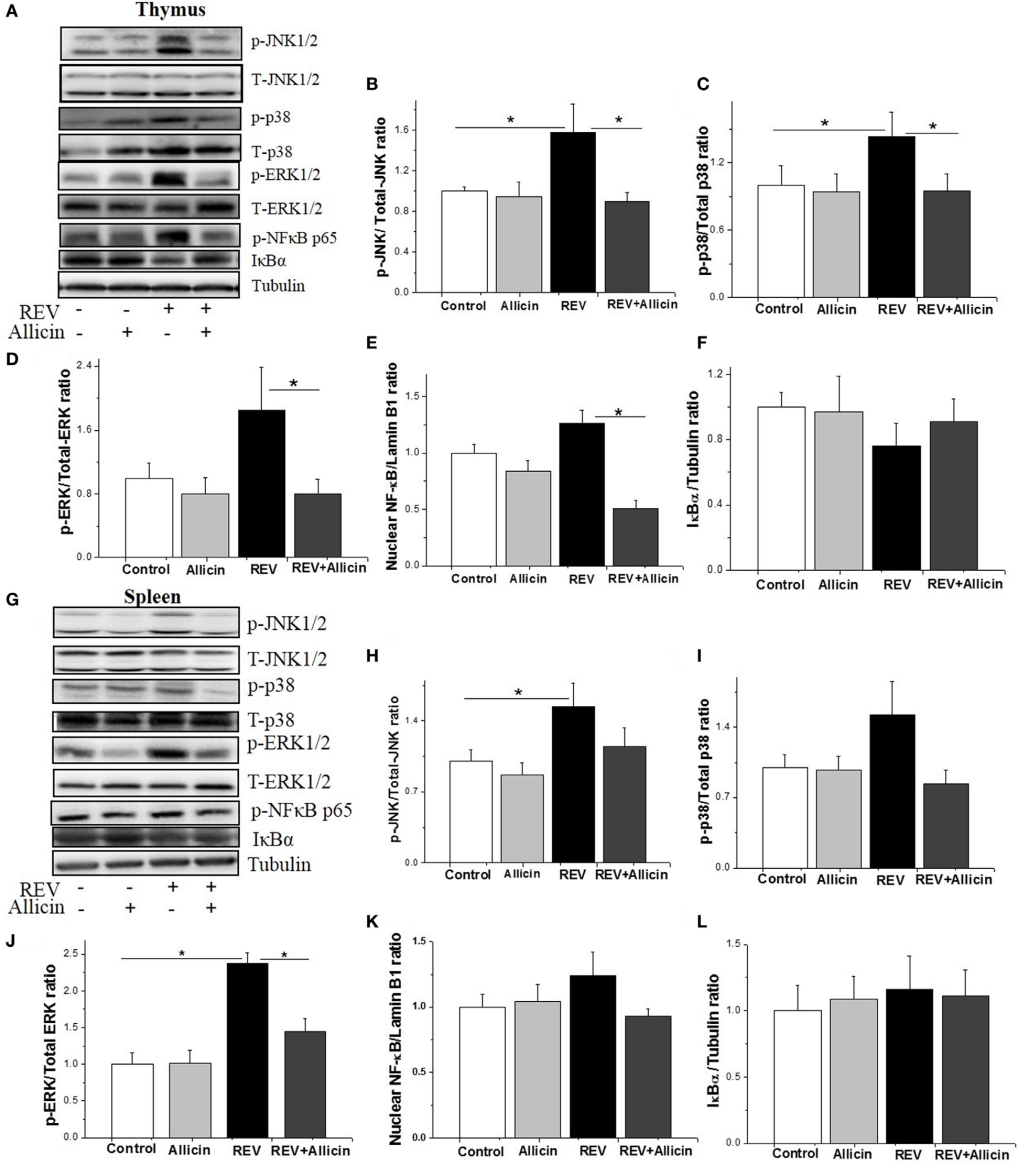
Allicin (300 mg/kg) suppresses the expression of mitogen-activated protein kinase (MAPK) and nuclear factor kappa B (NF-κB) activated by reticuloendotheliosis virus (REV) in thymuses. **(A,G)** Western blot analysis of MAPK and NF-κB in thymuses and spleens. The REV (lane 3) increases the expression levels of MAPK and NF-κB, and this increase was reduced by 300 mg/kg allicin (lane 4). **(B–F,H–L)** Relative intensities of MAPK and NF-κB. All data are presented as mean ± SE (*n* = 8) (folds of the control group). “*” denotes a statistically significant difference at *P* < 0.05 compared with the other groups.

Activation of MAPK signaling cascades, including the p38 MAPK, ERK, and JNK pathways, has been reported to increase antiviral activity. We further investigated the involvement of MAPK pathways in the REV infection and the anti-REV activity of allicin. In thymus, compared to control, Western blotting analysis revealed that the phosphorylation levels of JNK and P38 were significantly elevated by REV infection (*P* < 0.05, Figures 4A–C), while the phosphorylation level of ERK was increased by REV treatment compared with the allicin group (*P* < 0.05, Figure 4D). In REV-infected chickens, allicin supplementation restored the phosphorylation levels of JNK, P38, and ERK (*P* < 0.04, Figures 4B–D). The nuclear level of NF-κB p65, a downstream target of MAPK, was also increased (*P* < 0.05) by REV and decreased (*P* < 0.05) by allicin supplementation in REV-infected chickens (Figure 4E), indicating the activation of NF-κB signaling pathway by REV and the blockage by allicin treatment. In spleen, REV infection increased the phosphorylation levels of JNK and ERK, compared to control (Figures 4G–I). In REV-infected chickens, allicin supplementation inhibited the phosphorylation level of ERK (*P* < 0.05, Figure 4J), while had no detectable effect on JNK and p38 (*P* > 0.05, Figures 4H,I). Nuclear level of NF-κB was not altered by REV or allicin treatment in spleens (*P* > 0.05, Figure 4K). In addition, the protein level of IκB was not changed by either REV or allicin treatment in both thymus and spleen (*P* > 0.05, Figures 4F,L).

**Figure 5:**
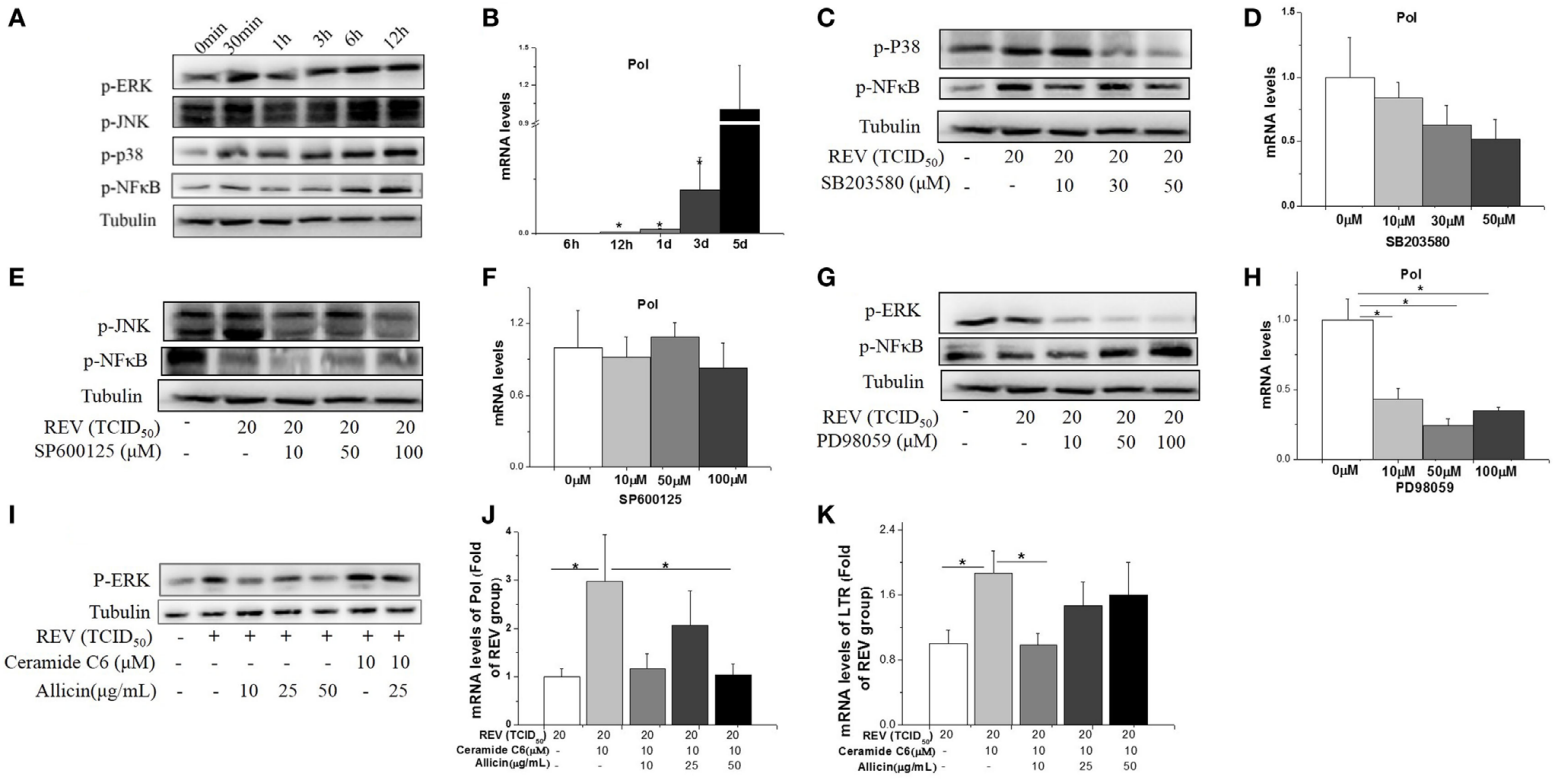
Allicin (300 mg/kg) inhibited the reticuloendotheliosis virus (REV) replication in lymphocytes isolated from spleens by reducing the phosphorylation of ERK. **(A)** Western blot analysis of mitogen-activated protein kinase and nuclear factor kappa B (NF-κB). **(B)** REV replication (folds of the expression of pol in 5 days). **(C,D)** Effect of P38 inhibitor on the expression of p38 and REV *pol* (folds of the expression of pol in 0 µM group). **(E,F)** Effect of JNK inhibitor on the expression of JNK and REV *pol* (folds of the expression of pol in 0 µM group). **(G,H)** Effect of ERK inhibitor on ERK and REV *pol* (folds of the expression of pol in 0 µM group). **(I)** Western blot analysis of REV and ERK activator (Ceramide C6) on ERK. **(J,K)** Effect of Ceramide C6 and allicin on REV replication (folds of the expression of pol and LTR in REV group). All data are presented as mean ± SE (*n* = 6). “*” denotes a statistically significant difference at *P* < 0.05 compared with the other groups.

We further investigated if MAPK and NF-κB pathway is involved in REV infection and the anti-infection effect of allicin. The lymphocytes separated from spleens of healthy chickens were used in *in vitro* experiment. The results showed that the phosphorylated JNK, p38, ERK, and NF-κB were increased after 30 min post REV infection (Figure 5A). REV pol gene was increased since 3 days post REV infection (*P* < 0.05, Figure 5B).

Cells pretreated with the JNK inhibitor (SP600125) and p38 inhibitor (SB203580) significantly (*P* < 0.05) decreased the phosphorylation of JNK, NF-κB (Figure 5E), and p38 (Figure 5C) separately, but had no significant effect on REV replication (*P* > 0.05, Figures 5D,F). In contrast, ERK/MAPK inhibitor (PD98059) treatment significantly (*P* < 0.05) inhibited the REV-induced phosphorylation of ERK (Figure 5G) and REV replication (Figure 5H). In contrast to the suppression effect of JNK and p38 inhibitors, the phosphorylation level of NF-κB was increased by ERK inhibitor treatment (Figure 5G).

We further detected the effect of ERK signal in REV replication. The REV activator (Ceramide C6) was employed to activate the ERK pathway. The result showed that Ceramide C6 enhanced the phosphorylation level of ERK (Figure 5I) and upregulated the expression levels of *pol* and *LTR* (*P* < 0.05, Figure 5K), compared with control and REV treatment. In contrast, the combined treatment of allicin with REV and Ceramide C6 decreased the phosphorylated ERK level (Figure 5I) and downregulated the expression level of *pol* at 50 µg/mL (*P* < 0.05, Figure 5J) and *LTR* at 10 µg/mL (*P* < 0.05, Figure 5K).

### Allicin Increases the Activities of Enzymic Antioxidants in REV-Infected Chickens

**Figure 6:**
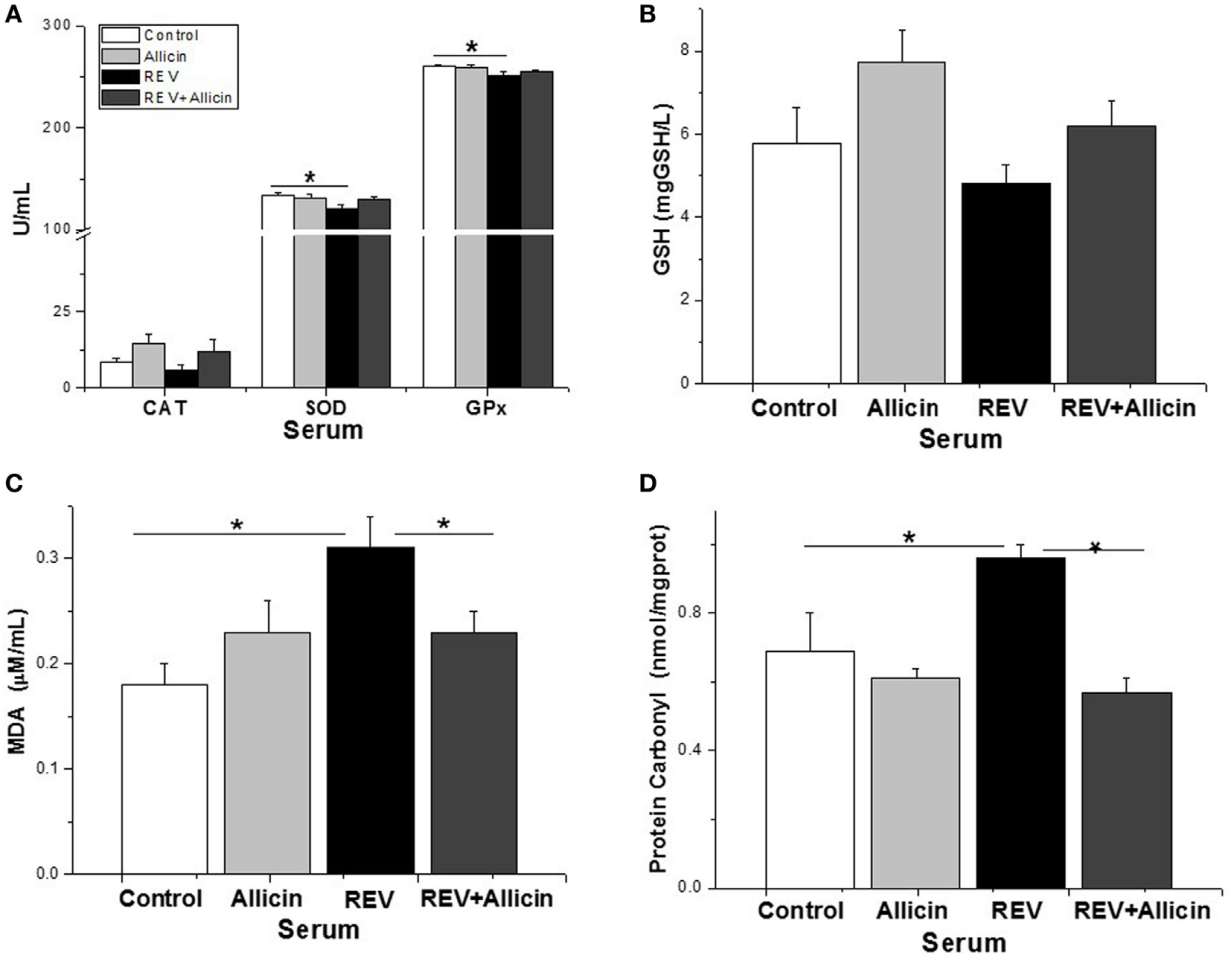
Allicin (300 mg/kg) increases the antioxidant capacity to defend against reticuloendotheliosis virus (REV) infection. **(A)** The activity of catalase (CAT), superoxide dismutase (SOD), and glutathione peroxidase. **(B)** The content of GSH. **(C)** The content of malondialdehyde (MDA). **(D)** The content of protein carbonyl. All data are presented as mean ± SE (*n* = 8) (folds of the control group). “*” denotes a statistically significant difference at *P* < 0.05 compared with the other groups.

Glutathione peroxidase, CAT, and SOD are important enzymes of the antioxidant system. The present study indicated that the serum activities of antioxidase including SOD (*P* < 0.05) and GSH-PX (*P* < 0.05) except for CAT (*P* > 0.05) were significantly decreased in REV-infected chickens compared to the control ones (Figure 6A). Unexpectedly, supplement of allicin had no statistically effects on the activities of SOD and GSH-PX (*P* > 0.05) in REV-infected chicks. Compared to control, the serum content of GSH was not significantly changed by REV infection (Figure 6B). MDA is an end product of lipid peroxidation that is considered harmful and may be responsible for cell death, causing tissue damage. The protein carbonyl content is a sensitive index of protein oxidative damage. Our results showed that the contents of MDA (*P* < 0.05, Figure 6C) and protein carbonyl (*P* < 0.05, Figure 6D) were significantly increased by REV infection compared with the control group. In contrast, we observed a significant reduction in the levels of MDA and protein carbonyl in REV-infected chickens treated with allicin (*P* < 0.05).

**Figure 7:**
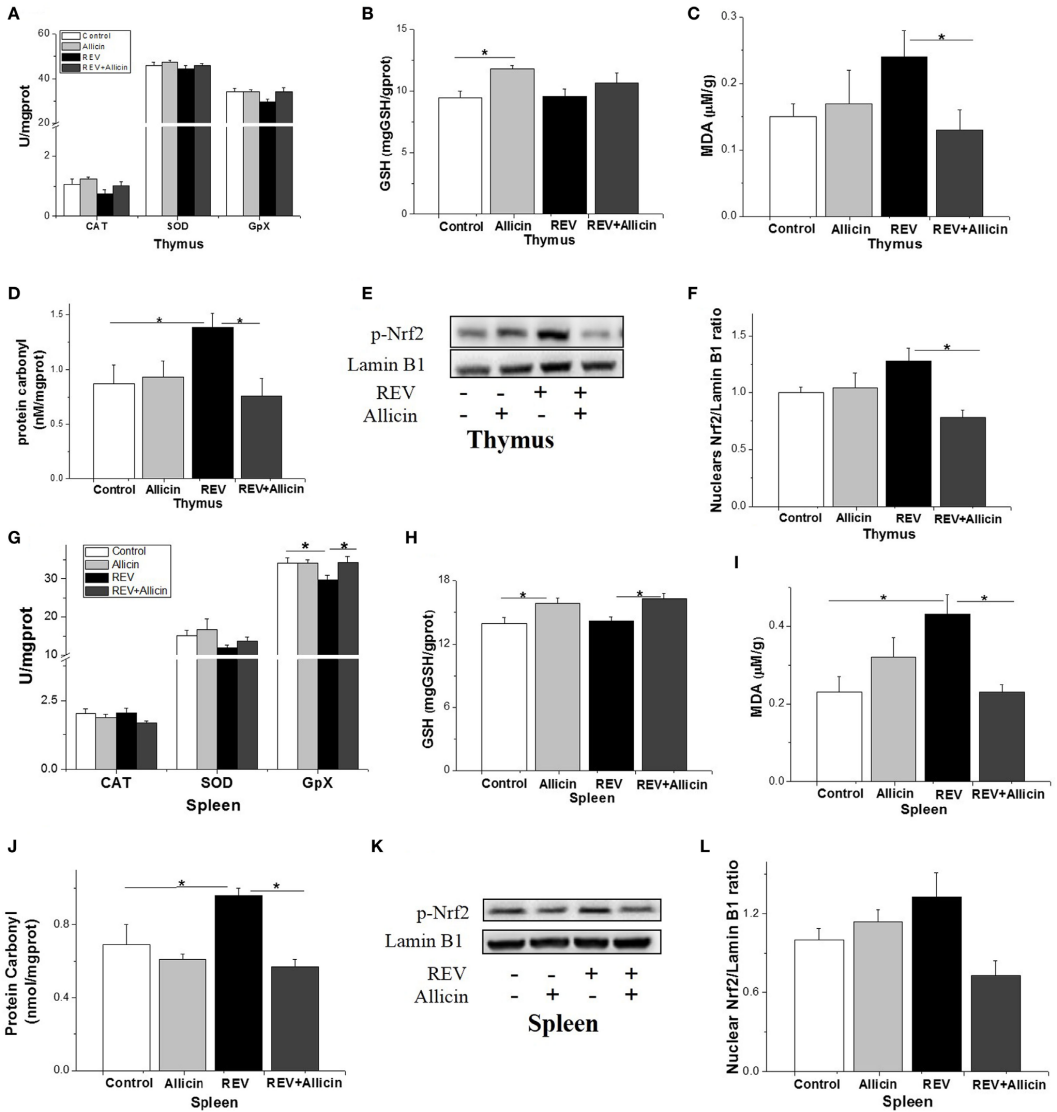
Allicin (300 mg/kg) alleviates the oxidative stress in reticuloendotheliosis virus (REV)-infected thymuses and spleens. **(A,G)** The activity of catalase (CAT), superoxide dismutase (SOD), and glutathione peroxidase. **(B,H)** The content of GSH. **(C,I)** The content of malondialdehyde. **(D,J)** The content of protein carbonyl. **(E,K)** Western blot analysis of nuclear factor erythroid 2 p45-related factor 2 (Nrf2) in thymuses and spleens. **(F,L)** Protein levels of Nfr2 to Lamin b1. All data are presented as mean ± SE (*n* = 8) (folds of the control group). “*” denotes a statistically significant difference at *P* < 0.05 compared with the other groups.

In thymus, the activities of SOD, CAT, and GSH-PX were not influenced by REV infection or allicin treatment (*P* > 0.05, Figure 7A). GSH level was increased by allicin supplementation (*P* < 0.05, Figure 7B). REV-infected chickens had higher level of protein carbonyl compared to control (*P* < 0.05, Figure 7D). In REV-infected chickens, allicin supplementation decreased MDA and protein carbonyl levels (*P* < 0.05, Figures 7C,D). The phosphorylated Nrf2 was increased by REV infection but suppressed by allicin treatment in REV-infected chickens (Figures 7E,F). In spleen, the activity of GSH-PX (*P* < 0.05) was decreased by REV infection and restored to normal level by allicin treatment (*P* < 0.05). However, there were no statistically detectable changes (*P* > 0.05) in SOD and CAT compared with the control group (Figure 7G). GSH level was increased (*P* < 0.05) by allicin supplementation and diminished by REV infection (*P* > 0.05, Figure 7H). REV infection upregulated while allicin declined the MDA and protein carbonyl concentrations (*P* < 0.05, Figures 7I,J). The phosphorylation level of Nrf2, however, was not changed by REV or allicin treatment (*P* > 0.05, Figures 7K,L).

## Discussion

Reticuloendotheliosis virus infection mainly results in runting syndrome, growth retardation, and immune suppression (1). It is reported that REV can be combined into a number of genomes of attenuated vaccines such as FPV and MDV and induce immunosuppression to result in great economic losses in poultry industry (4–6). However, there are no effective vaccines and drugs. In the present study, we successfully established REV-infection model, and the antivirus effect of allicin was evaluated. The present results demonstrated that allicin could alleviate the unfavorable influence of REV infection *via* blocking ERK/MAPK pathway, which brings valuable clues for clinic strategies in the prevention and control of REV infection.

### Allicin Alleviates the Immune Dysfunction Induced by REV Infection

In chickens, immune organs such as thymuses, spleens, and bursas are the sites where T- and B-cells differentiate. REV is known to attack the reticuloendothelial cells or lymphocytes to induce humoral and cellular immune suppression, inducing the abnormal development of immune organs and finally inducing immunosuppression of the host (3, 15). In the current study, REV infection caused atrophy of thymuses and bursas, indicating the impaired immune function. The enlargement of spleens in REV-infected chickens was in line with previous studies (3). Similarly, REV infection resulted in serious extravasation of spleens, and REV infection may induce lymphoma which can infiltrate spleen cells. In addition, REV can cause reticuloendotheliosis and induce abnormal hyperplasia of tissue cells (3). Allicin, the main active ingredient of garlic, has a function of immune regulation (13, 16). In the REV-infected chickens, allicin supplementation increased the weight of thymus and bursas and restored spleen weight to normal level (13). The results indicate that allicin could diminish the influence of REV on the development of immune organs in REV-infected chickens.

Cytokines play important roles in regulating innate immune responses and acquired immunity (2). Analysis of cytokine profiles will provide more information on the mechanism of immunosuppression caused by REV. High levels of pro-inflammatory cytokines such as IL-1*β* and IL-6 induce, amplify, and prolong the inflammation. MDV, another oncogenic virus in chicken, was reported to induce the overexpression of IL-1*β* and IL-6, inducing a Th1-type immune response (7). In this study, the upregulated expressions of IL-1*β* and IL-6 in thymus, spleen, and bursa indicate the evoked innate immune response and inflammation in REV-infected chickens. Infection with REV-CS resulted in a 10-fold increase in IFN-γ mRNA levels in 9–10- or 30-day-old birds (17). Chickens infected with SNV strain of REV showed three- to five-fold increased level of IFN-γ between 7 and 28 dpi as measured by Ag capture ELISA (18). In the present research, the SNV strain of REV also promoted the mRNA level of IFN-γ. However, I-IFN including IFN-α and IFN-β was downregulated by REV, consistent with previous report (2). TNF-α is a potent immunomodulator and pro-inflammatory cytokine that has been implicated in the pathogenesis of autoimmune and infectious diseases. It has been reported that the TNF-α system was activated during HIV-1 and J subtype avian leukosis virus (ALV-J) infection and the raised level increased with disease progression and degree of immunodeficiency (19). Since TNF-α has a strong antitumoral action (20), the upregulated expression of TNF-α post REV-A infection may correlate with tumor caused by REV (2). High level of TNF-α was observed post REV-SNV infection, which may result in severe damage of immune organs and immune dysfunction. IL-10 is an immunosuppressive cytokine and plays a role as a mediator of tumor regression (21). The upregulated expression of IL-10 in thymuses, spleens, and bursas suggests the increased susceptibility to concurrent or secondary infections by REV infection. The results indicated that REV infection disturbs the delicate balance in cytokine networks, which contributed to the increased susceptibility to concurrent or secondary bacterial or viral infections.

In previous study, allicin can inhibit the release of inflammatory cytokines and improve immunity (22). In ankylosing spondylitis mice, allicin significantly inhibited the secretion of TNF-α and IL-6 (10). The expression of IFN-γ was promoted by allicin treatment to suppress tumor growth and prolonged survival time (23). In accordance with the previous study, the present results revealed that allicin could reduce the release of pro-inflammatory cytokines mentioned above and increase the expression of I-IFN and IL-2, indicating that allicin could improve the REV-triggered decreased immune function.

Although the pathogenesis of REV is still not fully clarified, microarray data analysis has revealed that the genes related to cytokine–cytokine receptor interactions, metabolic processes, cell adhesion, and immune responses are closely linked to the immunosuppression induced by REV (15). Retroviruses can selectively trigger a series of innate immune responses through various PRRs (24). However, the nature of the exact innate sensors detectable for REV has remained elusive until now. TLR-3 is known to play a key role in the host response to virus infection by recognizing virus-derived dsRNA in intracellular vesicles to induce the production of IFN (25), whereas TLR-4 recognizes viral structural proteins on the plasma membrane (26). Our results showed that REV induces a significant increase in TLR-3 protein in thymus and mRNA level of TLR-4 in spleen. The results imply that chicken TLRs are associated with REV recognition. As reported, TLR ligands work additively *via* MyD88 to initiate the protective immunity in mice. The combined adjuvant acts *via* MyD88 can induce hemagglutinin-specific antibodies and protect mice against influenza virus challenge (27). Consistently, the expression of adaptor protein MyD88 was also overexpressed in REV-challenged chickens compared with that in controls. MDA5 is a RIG-I-like cytoplasmic sensor of dsRNA and certain RNA viruses, for the initiation of the IFN signaling cascade in the innate antiviral response (28). In this study, REV infection also induced a significant increase in the mRNA expression of MDA5, which can be activated by encephalomyocarditis virus and partially compensates to generate an IFN response (28). The result may imply that MDA5 is involved in the sensing of REV. However, allicin treatment decreased the TLRs and MDA5 expression induced by REV, suggesting that allicin may control REV-induced TLR-related signaling pathways, and these effects may contribute to its inhibitory effect on cytokine gene expression.

### REV Activated While Allicin Blocks ERK/MAPK Pathway during REV Infection

In mammals, MAPKs are activated upon pathogen challenge, leading to the expression of inflammatory cytokines and chemokines (29, 30). Previous studies have reported that hepatitis B virus core antigen infection activates the NF-κB, ERK, and p38 MAPK pathways, leading to the production of pro-inflammatory and regulatory cytokines in human monocytic THP-1 macrophages (31). Additionally, the ERK pathway is activated during ALV-J infection (32). In this study, p38, JNK, and ERK pathways were significantly activated by REV infection, which was probably associated with sustained inflammation (33). We further investigated the activation of p38, JNK, and ERK separately in the presence of REV in lymphocyte separated from spleens. The activation of p38 and JNK cannot increase the replication of REV, suggesting that p38 and JNK play a minor role in REV infection. Adversely, the activation of ERK promoted the replication of REV, while inhibition of ERK deceased the expression of REV-related genes, indicating that ERK pathway played an important role during REV infection.

Nuclear factor kappa B is considered one of the major transcription factors involved in pro-inflammatory gene regulation and is generally present in the cytoplasm as a heterodimer complex of p65/p50 subunits combined with the inhibitory protein IκB. Inflammatory stimuli induces the rapid degradation of IκB; subsequently, the free NF-κB molecules translocate into the nucleus, bind to target DNA elements, and activate the transcription of genes that encode proteins involved in inflammatory responses (34). In the present study, the nuclear expression of NF-κB in REV-infected chicks was elevated in thymus but not in spleen, showing the tissue specific response. However, REV infection showed no significant influence on the expression of IκB. The result indicated that activated NF-κB pathway in REV-infected chickens, which should be responsible for the upregulated expression of cytokines. The blockade of the NF-κB pathway has achieved some success to inhibit inflammation in murine models (35). Similarly, allicin treatment was shown to block the activation of NF-κB (36). Collectively, the result suggests that ERK/MAPK and NF-κB pathways are involved in REV-induced inflammation.

Allicin was reported to suppress the activity of NF-κB and inhibit MAPK pathways to alleviate inflammation in trinitrobenzenesulfonic acid-induced rats (37). In the present study, we found that allicin downregulated the phosphorylation of p38, ERK, and JNK in thymus and suppressed the phosphorylated ERK in spleen of REV-infected chickens, suggesting that allicin could block MAPK pathway during REV infection. In lymphocytes, allicin inhibited the activation of ERK and REV replication, suggesting that allicin may reduce the REV expression by inhibited the activation of ERK.

### Allicin Reliefs the Oxidative Stress Induced by REV Infection

Virus infection activated the macrophages and monocytes to release ROS and RNS, leading to tissue inflammation and oxidative stress (38). Antioxidant enzymes such as CAT, SOD, and GSH-PX play important roles in antioxidative systems. SOD converts O_2_ to H_2_O_2_, which is subsequently neutralized to water by CAT and GSH-PX (39). Previous reports showed that HIV infection reduced the activity of SOD and GSH-PX, damaged the oxidative defense system, and induced strong oxidative stress (40). REV infection increased the levels of MDA and protein carbonyl in serum, thymus, and spleen, indicating that REV infection induced oxidative damage in immune organs. The decreased serum SOD and GSH-PX activities and GSH-PX in spleen indicated the suppressed antioxidative enzymes during REV infection. The unobvious change in GSH in both serum and immune organ thymus and spleen suggests the unchanged antioxidative substance during REV infection. Collectively, the results suggest that weakened antioxidative enzyme should be responsible at least partially for the augmented oxidative damage during REV infection. Allicin has antioxidant effects to limit free radical damage (39). In HIV-infected individuals, the content of selenium in blood was decreased, but supplementation with allicin increased the content of selenium to increase the activity of GSH-PX, finally inhibiting viral replication induced by inflammatory factors (41). In accordance with the results mentioned above, allicin induced the elevation of GSH both in thymuses and spleens implied the increased antioxidant capacity. Similarly, allicin increased the activity of CAT, SOD, and GSH-PX in REV-infected chickens. MDA is considered a byproduct of lipid peroxidation, and protein carbonyl is a byproduct of protein oxidative damage (14). These findings indicated that REV infection may induce oxidative damage of the experimental chicks. Allicin has a direct antioxidant effect, acting as a scavenger of free radicals or inhibiting lipid peroxidation (4, 42). Allicin attenuates oxidative stress by reducing the levels of intracellular ROS and MDA and enhancing the glutathione/glutathione disulfide ratio (43). In line with the previous studies, the decreased MDA and protein carbonyl in serum, thymus, and spleen of REV-challenged chickens by allicin supplementation indicated the attenuated oxidative damage induced by REV. In REV-challenged chickens, phosphorylated Nrf2 was lower in the allicin group compared with REV-infected chickens, suggesting that Nrf2 pathway is not activated in the presence of allicin during REV challenge. We speculated that allicin supplementation decreased oxidative stress by directly reducing ROS formation (43). This speculation was supported by the observation that GSH concentration and antioxidant enzyme activity were not lower than that in health chickens.

In summary, the results showed that allicin treatment markedly reduced REV-induced immunosuppression. These results indicated that ERK/MAPK pathway is involved in the replication of REV. Allicin could alleviated REV-induced inflammation. Moreover, the antioxidant effect of allicin contributes to its antiviral function. The results shed a light on the pathogenic mechanism of REV and the anti-REV mechanisms of allicin, which may represent a therapeutic target for future development and discovery of anti-REV drugs.

## Ethics Statement

All animal disposal procedures were sanctioned by the Chinese Council on Animal Care and Institutional Animal Care and Use Committee of Shandong Agricultural University. All animal experiments were conducted according to the regulations and guidelines established by this committee and international standards for animal welfare.

## Author Contributions

LW designed the study, designed and performed experiments, analyzed, and interpreted the data, designed the figures, and wrote the manuscript. SS and HL participated in the design of the study and interpreted the data. XW, JZ, and HJ provided essential reagents and participated in analysis and interpretation of the data. All authors read and approved the final manuscript. All the authors critically reviewed, edited, and approved the final manuscript.

## Conflict of Interest Statement

The authors declare that the research was conducted in the absence of any commercial or financial relationships that could be construed as a potential conflict of interest. The reviewer CJ and handling editor declared their shared affiliation.
